# Effect of Nicotinic Acid (Vitamin B_3_ or Niacin) on the lipid profile of diabetic and non – diabetic rats

**DOI:** 10.12669/pjms.295.4193

**Published:** 2013

**Authors:** Talmeez Zeb Shah, Abdul Basit Ali, Saghir Ahmad Jafri, M.H. Qazi

**Affiliations:** 1 Dr. Talmeez Zeb Shah, Institute of Molecular Biology and Biotechnology, The University of Lahore, Lahore, akistan.; 2 Dr. Abdul Basit Ali, Institute of Molecular Biology and Biotechnology, The University of Lahore, Lahore, akistan.; 3 Prof. Dr. Saghir Ahmad Jafri, Institute of Molecular Biology and Biotechnology, The University of Lahore, Lahore, akistan.; 4 Prof. Dr. M.H. Qazi, Institute of Molecular Biology and Biotechnology, The University of Lahore, Lahore, akistan.

**Keywords:** Nicotinic acid, Lipid profile, Diabetic and non diabetic Hyper-cholesterolemic rats

## Abstract

***Objective:*** To determine the efficacy of nicotinic acid on the lipid profile of diabetic and non diabetic rats.

***Methods:*** This was an experimental study done at the Institute of Molecular Biology and Biotechnology, The University of Lahore, Pakistan between May 2010 to July 2010. Nicotinic acid was administered to a hypercholesterolemic group and a hypercholesterolemic + diabetic Group of Albino rats for 42 days and response to therapy was recorded on day 21 and day 42 of the experiment. Comparison among these two groups as well as three control groups was determined by Analysis of Variance (ANOVA) and differences were considered significant at (P<0.05). A total of 50 rats were included in the study.

***Results:*** Lipid profile of the hypercholesterolemic group as well as hypercholesterolemic + diabetic group as compared with the control groups showed highly significant improvement on the day 21 and day 42 of the experiment. The values of serum total cholesterol (TC), triglycerides (TG), low density lipoprotein (LDL) cholesterol and total lipids (TL) showed highly significant decrease whereas serum high density lipoprotein (HDL) cholesterol showed highly significant increase.

***Conclusion:*** Nicotinic acid is the most effective agent available in increasing HDL cholesterol and lowering serum TC, triglycerides (TG), LDL cholesterol and TL in hypercholesterolemic Diabetic and hypercholesterolemic non-diabetic Albino rats.

## INTRODUCTION

Niacin or nicotinic acid can serve as a wonder drug for correcting lipid metabolic disorders besides it functions as a B group vitamin. It is a water soluble vitamin and has since long been used to treat lipid disorders, which, if left untreated, would result in atherosclerosis and cardiovascular problems. It has remarkable HDL cholesterol raising capability. It also lowers the bad cholesterol, that, is very low density lipoprotein (VLDL) and LDL as well as TG and fatty acids.^1^ Biochemically, nicotinic acid is a derivative of Pyridine, a pyridine-3-carboxylic acid. The effects of nicotinic acid on the lipid profile were studied and demonstrated more than fifty years ago. But the mechanism of action has only been recently made clear.

In the past, the use of drug was discontinued since patients could not tolerate nicotinic acid therapy which caused very troublesome cutaneous flushing. However, it has been shown that the prolonged release nicotinic acid has reduced the incidence of flushing by about 75%.^2^

Contrary to the previous belief that nicotinic acid cannot be used in diabetics, evidence is now available which shows it has only mild effect on the fasting glucose and hemoglobin A_1c_ levels.^3^ Even these slight changes can be controlled by available oral hypoglycemics. It has been reported that with the use of this drug progression of atherosclerosis is decreased, cardiovascular events are reduced to a large extent in patients with diabetic dyslipidemia.^4^ In an earlier trial nicotinic acid had shown that the incidence of cardiovascular events is reduced by 90%. Accordingly, researchers have strongly recommended that the use of this highly effective and affordable medicine be promoted provided that the dose is gradually increased to avoid flushing. This should be accompanied by effective counseling in order to help patients to achieve maximum benefit from this important cardio protective agent.^5^

Little information is available from Pakistan on the use of this drug. The present study was undertaken to assess the response of nicotinic acid in hypercholesterolemic as well as hypercholesterolemic + diabetics in the environment of a developing country like Pakistan where most of the patients do not have the luxury to buy a drug like statins to control their lipid profile. Moreover, patients who do not find statins suitable because of its side effects can have an alternative treatment available. The experiments were carried out on rats because of its well defined genetic system which has high similarity with the human genetic system. 

## METHODS

It was an experimental study conducted over a period of 42 days from May 30, 2010 to July 11, 2010 at the Institute of Molecular Biology and Biotechnology, The University of Lahore. 

Response of nicotinic acid administration was seen for two different indications for which rats were divided into five different groups of 10 rats each. Albino rats (*Rattis Norvagicus)* with initial weights ranging from 240 to 300 grams were selected for this experiment. The rats were given standard laboratory diet which was isocaloric (2700 cal/kg feed) and isonitrogenous (21% crude protein) and were allowed free access to drinking water. The rats were fed for 42 days as follows:

Group 1: Control A: standard poultry diet only.

Group 2: Control B: standard poultry diet + 2 ml of olive oil daily administered orally with a dropper.

Group 3: Diabetic C: standard poultry diet + 2 ml of olive oil daily administered orally with a dropper. 

Group 4: Hypercholesterolemic: standard poultry diet + nicotinic acid 8.5 mg / 100 g of body weight in 2 ml of olive oil daily administered orally with a dropper.

Group 5: Hypercholesterolemic and diabetic: standard poultry diet + nicotinic acid 8.5 mg/100 g of body weight in 2 ml of olive oil daily administered orally with a dropper.

***Dose Preparation:*** Niacin tablets (500mg) manufactured by Upsher-Smith Laboratories, Inc. (Minneapolis, MN 55447 USA) were powdered. The oral dose 8.5mg/100g of body weight was calculated for every rat of group 4 and 5. It was prepared in 2 ml of olive oil, as prescribed by.^6^

The first reading of blood serum was taken on day one when groups 3, 4 and 5 had achieved diabetes and hypercholesterolemia. It was at this stage that the administration of nicotinic acid was started. The 2^nd^ reading was taken on the day 21. The 3^rd^ reading was taken on the day 42. One ml blood was collected from the coccygeal vein of tail of each rat after an overnight fast. Blood was centrifuged to separate serum within one hour after collection. Chemical analysis was done on each sample for blood sugar and serum lipids using enzymatic kits; (Cenix Diagnostics for blood sugar; HUMAN for HDL and for TC; RANDOX for TG and Bio Science for TL). LDL cholesterol was calculated according to Friedewald equation which is as follows: LDL-Cholesterol (mg/dl) = Total Cholesterol – HDL Cholesterol – Triglycerides / 5

- Diabetes was induced in Group 3 and Group 5 rats. Rats were fasted for 24 hrs before inducing diabetes since hyperglycemia has been reported to inhibit alloxan (Alloxan monohydrate, produced by pharmaceutical company Sigma, Germany) induction of diabetes. A single dose of 65 mg/kg body weight alloxan was administered intrapertioneally to the rats to induce diabetes. Diabetes was confirmed 10 days after alloxan injection with a glucose enzymatic kit by spectrophotometer. Rats with blood glucose level greater than 150 mg/dl were taken as diabetic.^7^
- The rats of Group 4 and Group 5 were made hypercholesterolemic by oral administration of 1% cholesterol powder in a dose of 1g/Kg of body weight for 10 days.^8^ The hypercholesterolemic condition was confirmed by detecting serum cholesterol on 11^th^ day, by using respective diagnostic kit and a level above 150 mg/dl was taken as hypercholesterolemic.
- The effects of nicotinic acid on the lipid profile of diabetic and non-diabetic rats were assessed. All values were expressed as mean ± standard deviation (SD) in descriptive statistics, distribution of variables were tested using Repeated measure Analysis of Variance (ANOVA) at (P<0.05). Statistical significance of differences between the two Control groups and the Diabetic C group on one hand and the two hypercholesterolemic study groups on the other hand were also evaluated by above test. All the data thus obtained was analyzed statistically by using repeated measure Analysis of Variance (ANOVA).^9^ We also provide P Value and F value.

## RESULTS

It was seen that after 42 days of treatment with Nicotinic Acid Total Cholesterol (TC) of group 4 decreased from 213 ± 3.02 mg/dl to 149 ± 3.86 mg/dl which is highly significant (P<0.05). Similarly, Total Cholesterol of Group 5 decreased from 217 ± 3.33 mg/dl to 161 ± 1.63 mg/dl which is also highly significant as (P<0.05.)

 The increase in HDL-cholesterol, was seen in both the Groups 4 and 5. In Group 4, the HDL-cholesterol increased from 29.4 ± 1.5 mg/dl to 57.5 ± 1.36 mg/dl in 42 days. Similarly, it increased from 21.6 ± 2.7 to 56.5 ± 1.9 mg/dl in Group 5 in 42 days. The results were highly significant as (P<0.05) in both the cases.

 LDL-Cholesterol also showed a highly significant decrease in both Groups 4 and 5 in 42 days as (P<0.05). Values of LDL-Cholesterol decreased from 135 ± 1.8 mg/dl to 67 ± 5.2 mg/dl in Group 4. Similarly, it decreased from 147 ± 5.72 mg/dl to 78 ± 4.02 mg/dl in Group 5.

 The decrease in Serum Triglycerides (TG) was from 225 ± 1.9 mg/dl to 131 ± 1.62 mg/dl in Group 4 and from 229 ± 5.6 to 128 ± 1.95 mg/dl in Group 5 in 42 days. Both the results were significant as (P<0.05).

 Serum Total Lipids (TL) also showed highly significant decrease (P<0.05) in both the Groups 4 and 5. Serum Total Lipids (TL) decreased from 982 ± 22.7 mg/dl to 449 ± 34 mg/dl in Group 4 and it decreased from 997 ± 10.4 mg/dl to 548 ± 48.4 mg/dl in Group 5 in 42 days of nicotinic acid treatment.

 Similarly, interaction of Groups and days also showed highly significant values as (P<0.05). The changes in values of Group 4 and 5 as compared to Group 1, Group 2 and Group 3 were highly significant.

## DISCUSSION

The descriptive statistics of mean serum levels (mg/dl) of TC, TG, LDL-C and TL showed significant decrease in hypercholesterolemic non diabetic and hypercholesterolemic diabetic groups. Although the improvement in lipid profile in hypercholesterolemic diabetic rats showed a very slight lag yet the results are marvellous. These results are consistent with previous studies on non – diabetic dyslipidemic patients^10^ as well as studies on diabetic dyslipidemic patients.^11^ Several studies have shown that nicotinic acid does not increase the synthesis of HDL-C but inhibits the selective uptake and degradation of apoprotein A by the liver. In the process Cholesterol ester in the HDL particle is taken up by the liver and apoprotein A is thrown back into the circulation which is the carrier protein of HDL particle. As such, more carrier protein is available for binding to HDL. As a result, HDL is available in circulation for longer periods and in larger quantity.^12^ In our study an increase in HDL was seen both in hypercholesterolemic non-diabetic and hypercholesterolemic diabetic rats. This is in consistence with a study where results of nicotinic acid administration to a group of rats was compared with a control group as well as a group which received 1/M dexamethasone and another group which received dexamethasone + nicotinic acid. Increase in serum HDL was highest in the group which was given nicotinic acid. There was also a decrease in serum total cholesterol as well as in serum LDL to the level even lower than the control group. Moderate fatty liver changes were seen in one third of rats on dexamethasone. Rats in control group and the group receiving nicotinic acid and the group receiving dexamethasone + nicotinic acid did not show signs of fatty changes.^13^ This is in agreement with the study called Assessment of Diabetes Control and Evaluation of the Efficiency of Niaspan Trial (ADVENT), carried out on diabetic patients.^14^ Similar results were obtained in another study on human beings designated as: The Arterial Biology for the Investigation of the Treatment Effects of Reducing Cholesterol (ARBITER).

An increase in HDL makes the reverse cholesterol transport mechanism very effective by taking up increased amount of cholesterol from the tissues which is then esterified by the enzyme phosphotidylcholine cholesterol acyl transferase (PCAT) and deposited in the core of HDL particles.^15^

Furthermore, it is known that the transport of cholesterol from macrophages in plaques to HDL is carried out with the help of a transporter called ATP binding cassette protein (ABCG-1). HDL particles transport cholesterol directly to the liver; or by binding cholesterol to VLDL, or Intermediate Density Lipoprotein (IDL) or LDL with the help of cholesterol ester transfer protein (CETP) and then to the liver. The liver either secretes the cholesterol in the bile or in the form of bile salts. Some of it is reabsorbed through the enterohepatic circulation. The net result is a decrease in serum total cholesterol.^16^

Data obtained in the present study is supported by the findings of previous workers who showed significant decrease in triglycerides level in rats made hypercholesterolemic with alcohol.^17^ Similar results have been reported in the study called Assessment of Diabetes Control and Evaluation of the Efficiency of Niaspan Trial (ADVENT).^18^

Nicotinic acid binds to its receptors Human Macrophage 74 A (HM74 A) / G Protein Coupled Receptor 109 A (GPR109 A) in adipocytes.^19^It is a G-protein coupled receptor. The ligand (nicotinic acid) recruits the inhibitory G protein coupled receptor. As a result there is decrease in cyclic Adenosine Monophosphate (cAMP) leading to decrease in activity of protein Kinase A. This in turn results in a decreased activity of lipase; as a result, the breakdown of triacylglycerols (triglycerides) into fatty acids and glycerols is inhibited leading to a decrease in mobilization of fatty acids from adipocytes. Consequently, there is a decrease of substrate (fatty acids) available to produce very low density lipoproteins (VLDL) in the liver. Nicotinic acid also decreases the synthesis of triacylglycerols (triglycerides) in the liver by inhibiting the enzyme diacylglycerol acyltransferase 2. Since triacylglycerols (triglycerides) are not available in liver, therefore, very low density lipoproteins (VLDL) are synthesized in liver in lesser amounts (triacylglyerides are incorporated in VLDL_s_ and carrier proteins of these particles are apoprotein B which are also synthesized in liver). Since very low density lipoproteins are circulating in lesser amounts therefore, the excess of their carrier proteins apoprotein Bs are degraded in liver). Lesser circulating VLDLs convert into lesser number of circulating low density lipoproteins LDLs. Very low density lipoproteins carry triglycerides contained in them, therefore, the amount of triglycrides is decreased in blood as a result of nicotinic acid administration.^20^ In the present research the low density lipoprotein cholesterol levels of both the group 4 and 5 showed highly significant decrease (P<0.05). The result is consistent with a previous study as well as the study called The Arterial Biology for the Investigation of the Treatment Effects of Reducing Cholesterol (ARBITER).^21^ It also showed correlation with the study called Prospective cardiovascular Munster study (PROCAM).^22^

Similarly, highly significant decrease in total lipid values was seen in response to the nicotinic acid administration (P<0.05). It was in correlation with the study conducted in 1964^23^ carried out on human beings. The decrease in Total lipids occurs in consequence to the cumulative effect of the changes in the lipid panel. 

The beneficial effects on the atherosclerotic changes as a result of prolonged administration of nicotinic acid to diabetic patients with dyslipidemia outweigh the modest and transient effects on levels of blood glucose. Provision of guidance has been made by, consensus clinical panels that includes the American Diabetes Association, American Heart Association, the National Cholestrol Education Program (NCEP) and adult treatment Panel-iii (ATP-III), for the proper and safe use of nicotinic acid. The effects of nicotinic acid given as a monotherapy or combined with statins were analyzed. The sugar levels in patients with disturbed lipid profile (both diabetics and non-diabetic) were studied. When nicotinic acid was given alone in a dose less than or equal to 2500 g/day or combined with statins, a rise of 4% -5% in fasting glucose level was seen and an increase of less than 0.3% in Hb A_1c_ level was seen. The changes were not significant. Moreover, the changes were mild, temporary and the values reverted back to the values which were noted at the start of the treatment. The small increase seen could be controlled by changes in oral hypoglycemic agents. There has been decrease in incidence of cardiovascular problems vs placebo when nicotinic acid has been given for prolonged periods of time. This has been seen in 6 years myocardial infarction, 6 years coronary artery disease death and 15-years all cause mortality rates (with or without metabolic syndrome). Treatment was stopped and patients were followed up for 9 years. It was found that cardiovascular risks were significantly reduced. There was a great decrease in cardiovascular problems. Diabetes Mellitus is not a contraindication for the use of nicotinic acid in treating dyslipidemia. Assessment of glucose level is recommended at the beginning of treatment as well as when the dose of nicotinic acid is increased. Changes in lifestyle are recommended as well as other initiatives should be taken to improve the glucose levels before the nicotinic acid therapy is begun in individuals with Hb A_1c_ of less than 8.0%. ^24^^,^^25^

Flushing is the main side effect. Other side effects are nausea, itching, diarrhea, decreased glucose tolerance, hypeuricemia and hyperhomocysteinaemia. Increased levels of liver enzymes, cholestasis and hepatocellular injury has been seen. Toxicity is dose related. Sustained release formulation is associated more with hepatotoxicity than the immediate release nicotinic acid and the extended release nicotinic acid. There should be a control of choice of nicotinic acid preparation, as well as its dose, so that its side effects are minimum. Switching on from one type of NA to the other, should not be done without a physician’s advise as switching over can do severe damage to the liver.^26^

The frequency of flushing, the main side effect of treatment, is reduced by about 75% with prolonged release nicotinic acid.

**Table-I:** Effect of nicotinic acid on lipid profile of hypercholesterolemic, non diabetic and hypercholesterolemic, diabetic rats

*Parameters*	*Group 1* *(Control A on feed only)*			*Group 2* *(Control B on feed + Olive Oil)*			*Group 3* *(Diabetic C on feed + Olive Oil)*			*Group 4* *(Hypercholesterolemic)*			*Group 5* *(Hypercholesterolemic and Diabetic)*			*P- statistics & P-value*			*F-statistics & F-value*		
	Day 1	Day 21	Day 42	Day 1	Day 21	Day 42	Day 1	Day 21	Day 42	Day 1	Day 21	Day 42	Day 1	Day 21	Day 42	Groups & P-value	Days & P-value	Interaction of Groups & P-value	Groups & F-value	Days & F-value	Interaction of Groups & F-value
Total cholesterol (mg/dl)	142±1.7	141±1.8	142±1.7	146±2.8	144±1.96	142±2.1	148±0.96	148±1.03	147±0.98	213±3.02	165±4.47	149±3.86	217±3.33	171±1.88	161±1.63	0.000	0.000	0.000	983.0	4422.7	1444.4
HDL-cholesterol (mg/dl)	48±1.4	49±1.9	48.23±1.16	48±1.8	47.8±1.8	47.8±1.48	44±1.04	43.4±1.19	42±1.88	29.4±1.5	53.7±1.7	57.5±1.36	21.6±2.7	51.6±2.6	56.5±1.9	0.000	0.000	0.000	25.74	4004.341	1838.649
LDL-cholesterol (mg/dl)	70±3.16	68±5.5	70±.3.04	70±4.0	72±6.6	69±3.31	75±0.95	74±1.16	74±1.6	135±1.8	79±4.22	67±5.2	147±5.72	94±3.10	78±4.02	0.000	0.000	0.000	238.6	7105.71	2508.36
Triglycerides (mg/dl)	132±5.14	132±4.33	132±5.2	132±3.9	132±4.24	130±4.4	130±31.2	138±1.65	138±1.42	225±1.9	169±4.5	131±1.62	229±5.6	166±3.6	128±1.95	0.000	0.002	0.000	432	8798.15	3119.64
Total Lipids(TL)	546±3.78	496±157	548±2.19	551±3.47	550±3.32	548±3.87	557±2.70	555±3.36	554±3.56	982±22.7	622±11.03	449±34	997±10.4	786±11.3	548±48.4	0.000	0.000	0.000	172.22	2088.58	776.27

Repeated measure ANOVA. Significant value (P value) P< 0.05 Overall significant difference in serum Total Cholesterol (TC), serum High Density Lipoprotein (HDL), serum Triglyceride (TG), serum Low Density Lipoprotein (LDL) and serum Total Lipids (TL) mg/dl was observed in all the groups as compared to the two Control Groups as well as the Diabetic C Group.

## CONCLUSION

The present research work showed that the effect of nicotinic acid on the lipid profile of diabetic and non-diabetic rats was highly significant. Nicotinic acid decreased the levels of serum total cholesterol, serum Triglycerides, serum low density lipoprotein cholesterol and serum Total lipids and it increased the high density lipoprotein cholesterol in both the diabetic as well as non-diabetic rats. 
